# On the Commoditization of Artificial Intelligence

**DOI:** 10.3389/fpsyg.2021.696346

**Published:** 2021-09-30

**Authors:** Abdullah A. Abonamah, Muhammad Usman Tariq, Samar Shilbayeh

**Affiliations:** Abu Dhabi School of Management, Abu Dhabi, United Arab Emirates

**Keywords:** artificial intelligence, AI commoditization, AI business value, AI strategy, AI operations

## Abstract

As artificial intelligence's potential and pervasiveness continue to increase, its strategic importance, effects, and management must be closely examined. Societies, governments, and business organizations need to view artificial intelligence (AI) technologies and their usage from an entirely different perspective. AI is poised to have a tremendous impact on every aspect of our lives. Therefore, it must have a broader view that transcends AI's technical capabilities and perceived value, including areas of AI's impact and influence. Nicholas G. Carr's seminal paper “IT Does not Matter (Carr, 2003) explained how IT's potential and ubiquity have increased, but IT's strategic importance has declined with time. AI is poised to meet the same fate as IT. In fact, the commoditization of AI has already begun. This paper presents the arguments to demonstrate that AI is moving rapidly in this direction. It also proposes an artificial intelligence-based organizational framework to gain value-added elements for lowering the impact of AI commoditization.

## Introduction

Artificial intelligence is a technological concept in operational management, philosophy, humanities, statistics, mathematics, computer sciences, and social sciences. Artificial intelligence aims to create computers or machines to carry out jobs that generally need human intelligence. The sub-discipline of artificial intelligence is machine learning, which then directs to statistical learning. Artificial intelligence is a branch of computer science that allows the machine to mimic human intelligence and execute tasks that humans can perform more efficiently. This term may also be applied to any machine that exhibits traits associated with a human mind, such as learning and problem-solving. This section is about how AI has developed over the years and about many important discoveries and inventions that have brought us to where AI is today.

In 1947, Alan Turing raised a life-changing yet straightforward query, “Can machines think?” Turing gave quite possibly the earliest public lecture (London, 1947) to mention computer intelligence, saying, “What we want is a machine that can learn from experience,” and that the “possibility of letting the machine alter its instructions provides the mechanism for this.” (Britannica, 2021). Decades later, scientists demonstrated that computers could, in fact, exhibit some form of intelligence. In the midst of the 1950s and 1970s, the computer industry discovered its foothold when computers began operating faster, became more approachable, and were less costly. An article in 1970 Life Magazine predicted that machines would shortly have a similar intellect to human beings in only 3–5 years. Still, extensive evolution in storage adequacy and computing capacity was required for that to take place. During the 1980s, there was an evolution of two effective techniques. The first one was the “expert system,” which imitated human's aptitude to make decisions. Computers started to utilize reasoning depended on “rules” - an “if-then/else” procedure used to respond to queries. The second was “machine learning,” which made computers learn through experience. In 1997, Dragon Systems made and adopted software for natural language speech recognition on Windows. In the 2000s, speed and storage options such as “cloud, catapulting the utilization of computers into the mainstream,” became more widespread providing artificial intelligence with its turn in the spotlight. Recently, advancements in artificial intelligence technologies have accelerated at a very rapid pace. The acceleration may be explained by three significant industry developments (Hildt, 2019).

- **“Graphics Processing Units (GPU)”:** Requirement propelled by the gaming and video world produced enhanced and less costly GPUs. In addition, these GPUs have increased the processing power of AI algorithms.
- **“Big Data and Machine Learning”:** Artificial intelligence uses machine learning algorithms to perform data analytics by building learning models to be used in “intelligent” ways. The learning models are based on the idea that machines can learn from data, identify patterns, and predict future states that help in decision-making with little human intervention.
- **“Deep learning”:** A subset of machine learning that can learn from the data without human intervention. For example, in classical machine learning (non-deep), machines need human interventions to label the unlabeled data. On the other hand, “deep” machine learning can leverage labeled datasets to inform its algorithm, but it does not necessarily require a labeled dataset to enable the unsupervised machine to train without any interventions.

It is widely acknowledged that AI can perform human-similar tasks with some level of intelligence, such as understanding verbal communication, driving cars, and distinguishing pictures. However, in comparison with human intelligence and understanding AI, the following section will shed light on three AI types.

### Types of Artificial Intelligence: ASI, AGI, ANI

There are three types of artificial intelligence: ASI (artificial super intelligence), AGI (artificial general intelligence), and ANI (artificial narrow intelligence).

- **Artificial Superintelligence:** This is a hypothetical ability of an intelligent agent to possess intelligence substantially exceeding that of the brightest and most gifted human minds. Currently, it is not technologically possible to produce machines that possess superintelligence properties.
- **Artificial General Intelligence (AGI):** This is the hypothetical ability of an intelligent agent to understand or learn any intellectual task that a human being can perform. Currently, it is a major focus of much artificial intelligence research. However, there is no existing intelligent agent that possesses the AGI properties (ref).
- **Artificial Narrow Intelligence (ANI):** ANI, also known as “weak” AI, is the most common today. Narrow AI can perform a single task—whether it is driving a car, playing chess, or recognizing spoken or written words. ANI systems are designed to focus on their tasks in real-time. With continuous learning from their environment, they build knowledge over time and become experts in performing their assigned tasks. However, these systems cannot perform tasks outside the single-task environment that they are designed for.

Artificial narrow intelligence is the most coherent kind of artificial intelligence to be utilized by most people. The following are some common examples of artificial narrow intelligence:

- **“Self-driving cars”:** A self-driving car, also known as an autonomous vehicle, driverless car, or robo-car, is a vehicle that is capable of sensing its environment and moving safely with little or no human input. Self-driving cars combine a variety of sensors to perceive their environment. These sensors include radar, lidar, sonar, GPS, odometry, and inertial measurement units. Advanced control systems interpret the sensors' data to identify appropriate navigation paths and obstacles and relevant signage.
- **“Voice assistant devices”:** A voice assistant is a digital assistant that uses voice recognition and natural language processing to listen and respond to verbal commands. Voice assistant devices are easy to use using voice-activated commands. From playing music to scheduling appointments, voice assistant devices make daily tasks easier. Some of these devices enable you to monitor your house on your smartphone, turn the lights on with a simple command, and access all your smart devices using just your voice. Alexa, Siri, and Google Assistant are the most common examples of these voice assistant devices. Voice-powered devices rely on artificial intelligence technologies to perform their voice recognition functions.
- **Robotics:** An AI application that can perform some tasks that need some level of intelligence, such as sensing obstacles and changing the path accordingly. It can be used for carrying goods in factories, cleaning offices, and inventory management.

Artificial general intelligence aims to advance artificial intelligence one step ahead, where machines can perform tasks at the human intelligence level. To achieve that goal, artificial general intelligence automata must pass a series of tests. It begins with the Turing Test, which Alan Turing originally designed in 1950. The Turing Test is a test of a machine's ability to exhibit intelligent behavior equivalent to or indistinguishable from a human being. If a machine gets a 70% or higher score, it is considered an artificial intelligence agent. The second test of artificial general intelligence's efficacy is done through the Coffee Test. This test asks the intelligent agent to get into a home environment, prepare coffee, and master the art of brewing it. Next, the College Robot Test requires the AGI robot to enroll in college and successfully pass all classes (Goertzel, 2017). Finally, the robot can appear in an Employment Test, where it has to clear a vocational test, including writing and driving exams (Keyes et al., 2021).

As mentioned above, AI capabilities are achievable by embedding some human intelligence. The following section will highlight different human-like intelligence forms and concentrates on learning as one of the most notable.

### Artificial Intelligence Learning Approaches

Artificial intelligence, defined as the machine's ability to mimic the human brain by performing tasks, needs some intelligence (Abbass, 2019). It includes learning, reasoning, problem-solving, and perception. Learning is the machine's ability to conclude or memorize knowledge without this information being fed into it. Human learning is distinguished into different forms. The simplest one is trial and error. However, the human brain can learn in a more complicated way. Similarly, machines are designed to learn using different learning approaches. These are machine learning (ML), deep learning (DL), and reinforced learning (RL).

- Machine learning is a subset of AI that involves techniques that enable machines to learn from the given data for pattern detection and future prediction (Agrawal et al., 2019).
- Deep learning is a subset of machine learning that makes a machine observe patterns and classify information, letting it “think” in a more advanced complicated way without the need for any human interventions.
- Reinforcement learning is similar to deep learning except that, in this case, machines learn through trial and error using data from their own experience.

Below is a close look at some artificial intelligence technologies and how they perform organizations' tasks.

- A “machine learning platform” can utilize information from various data sources- such as training and development tools, together with other algorithms to forecast and sort information (Bauguess, 2017).
- Deep learning is a machine learning technique that utilizes pattern classification and recognition to function with large data sets.
- Neural networks are machine learning techniques, which use statistical algorithms designed according to neuron behavior in the human brain.
- Cognitive computing is a kind of computing that utilizes high-grade understanding and reasoning. It is not contemplated as machine learning as it uses various artificial intelligence technologies to extract outcomes.
- Computer vision allows computer systems to function and act as a human eye. It examines the condition of digital pictures and videos to generate symbolic and numeric information for decision-making procedures.
- Natural language generation involves generating text from numeric characters. Organizations mainly utilize this procedure for reports, customer service, and business intelligence summaries.
- Graphical processing units (GPUs) are a division of an electronic circuit that amplifies picture formation on a display device. GPUs are essential for artificial intelligence to function successfully.
- Internet of Things (IoT) is a network of inter-connected devices that produce and share data, such as medical devices, smart speakers, appliances, and wearable technology. Artificial intelligence relies on these devices' data to make significant business intelligence decisions.
- Advanced algorithms are complicated algorithms that are continuously being generated and combined to furnish present intelligent processing.
- An application programming interface (API) is a technology that organizations utilize to acquire artificial intelligence services. Similarly, artificial intelligence uses data flows to support firms to make sense of data to help in organizational measures (Lawless et al., 2019).

Today, AI has become a mature technology and an increasingly important part of the modern fabric of life. AI is already deployed in different application domains.

The paper is organized as follows: In section Literature Analysis, the summary of the AI historical background emphasizing AI advantages and applications is provided. Section AI Commoditization discusses AI commoditization and presents some simple recommendations on “how to utilize AI to attain competitive advantages.” Section Are we ready for AI? answers the question: “Are we ready for AI?” and in section Summary of Findings and Conclusion, conclusions are provided.

## Literature Analysis

In recent years, there has been an abundance of research articles published in the area of AI. In the following section, we briefly present how AI has evolved during recent years:

### The Evolution of Artificial Intelligence

Artificial intelligence has been shown to be useful in fulfilling the following requirements (Davenport and Ronanki, 2018). A paper published in 1955 referred to a famous economist who wrote in 1828 regarding the probability of motor cars as replacements for horses: “Nevertheless no machine will ever be able to perform what even the worst horses can—the service of carrying people and goods through the bustle and throng of a great city.” People could never have imagined self-driving cars, intelligent mobiles, video calls, intelligent robots, pilotless airplanes, and supercomputers. Nevertheless, artificial intelligence that would have been considered science fiction <190 years ago are now available in today's era, and some, like self-driving motor cars, will most probably be in extensive use within the next 5 years (Katz, 2017). The challenge is to attempt to forecast future technologies based on artificial intelligence without repeating the errors of similar myopic scholars, who could not understand the significant computational evolution of the latest technologies (Dignum, 2019). There are two perceptions to be made. First, 190 years is a short period by ancient standards, and in the period, the world went from horses that were the most significant source of transportation to self-driving motor cars and from slide rules and abacuses to intelligent devices in our pockets (Roff, 2019). Secondly, the time frame between technological evolution and practical, general use is continuously being decreased. For example, there were more than 200 years from when Newcomen initiated the first ”workable steam engine“ in 1707 to when Henry Ford manufactured a dependable and cost-efficient motor car in 1908. It took more than 90 years between the time electricity was initiated and its general use by companies to enhance factory productivity. There were 20 years, non-etheless, between, ENIAC, the first computer ever, and the 360 system of IBM that was mass-produced and was budget-friendly for small business organizations. While it took 10 years from 1973 when Dr. Martin Cooper made the first mobile call through a handheld device and its public inauguration by Motorola.

The grand and most swift development started with smartphones when they first emerged in 2002. Smartphones have witnessed tremendous progress, with the latest versions consisting of significant enhancements every year by Samsung, various Chinese companies, and Apple (Villaronga et al., 2018). Smartphones, in addition to their technical features, now integrate the characteristics of artificial intelligence. These include speech recognition, furnishing personalized information in spoken language, finishing words during text typing, and various other features that need embedded artificial intelligence, offered by a pocket computer considerably smaller than a packet of cigarettes. The development has gone from intelligent computers to intelligent machines and toward programs based on artificial intelligence. A thermoregulator is a simple mechanical device that exhibits some primary but valuable intelligence that makes temperature constant at some preferred predetermined level (Haibe-Kains et al., 2020).

From digital computers to artificial intelligence tools: The Intel Pentium Microprocessor, launched in 1993, integrated music capabilities and graphics and unlatched computers to many cost-effective applications expanding more than data processing (Dunjko and Briegel, 2018). These technologies signal the start of a new phase that now involves smart personal assistants recognizing and responding to natural languages, robots capable of seeing and performing an array of smart operations, self-driving motor cars, and a range of other abilities close to that of human capability. The technology optimists determine that in <26 years, computers will have shifted from calculating 0 and 1 digits to using advanced neural network algorithms that allow the speaking of natural languages, vision, and understanding.

Tech optimists believe there is no doubt that in the next 21 years, augmented artificial intelligence technological advancement will lead to a leap in deep learning that emulates the way youngsters learn, instead of arduous guidance by custom-made programs directed for particular applications that are dependent on logic, decision trees, and if-then logic (Galbusera et al., 2019). For example, DeepMind depends on a neural program using deep learning that comprehends by itself how to play various Atari games, like Breakout, or better than humans, without detailed guidance for doing it, but by playing a dozen games and revamping itself every time. The program distinctly instructed AlphaGo that beat Go champion Lee Sodol in 2016 (Luckin, 2017). In addition, however, it will develop a new project base to understand how to play Starcraft, a complex game dependent on long-term plans and robust skillful decisions to stay ahead of the rival, which DeepMind plans to be its next target for progressing deep learning. Deep learning is a concept that seems to be the leading edge of research and funding attempts to enhance artificial intelligence, as its success has created a burst of activity in capital funding that gave more than $1.5 Billion to 125 projects for start-ups in the first quarter of 2019, in comparison to 31 projects in a comparable quarter of 2017 (Hall and Pesenti, 2017).

Google had five deep learning projects underway in 2019. Today it is continuing more than 4,000, according to their representative, in all its significant sectors, involving Gmail, self-driving cars, Android, YouTube, translation, and maps. IBM's Watson system utilized artificial intelligence, but not deep learning, when it defeated the two jeopardy champions in 2011. However, there has been a boost in all of Watson's 35 constituent services due to deep learning (Semmler and Rose, 2017). Shareholders who were not aware of deep learning 7 years ago today are considerate of start-ups that do not integrate AI into their programs (Cabitza et al., 2020).

For survival, it is necessary to develop advanced software applications to keep away from menus by integrating natural-language processing and clicking deep learning. How distant can deep learning go? According to tech optimists, there are no restraints for three causes (Leslie, 2019). The first one is, as development is accessible to everyone realistically to use through Open Source software, researchers will focus their attempts on new, stronger algorithms resulting in additive learning. The second one is that deep learning algorithms will be proficient in recollecting what they have learned and implementing it in the same way but in distinct situations (Sejnowski, 2020). Last and equivalently vital, in the future, intelligent computer systems will have the capability to develop new software by themselves, at first maybe not so advanced ones, but enhancing with time as learning will be integrated as a segment of their capabilities. Non-biological intelligence is expected to match the extent and refinement of human intelligence in almost a quarter of a century. This event, known as the “singularity,” is estimated to happen by 2045; it will bring the emergence of a new community to outmatch biological limits and boost our creativity (Corea, 2017). There will be no difference between machine and human, virtual reality, and human reality in this new era. For some individuals, this forecast is astonishing, with wide-ranging inferences should they become a reality (Galanos, 2018).

### Real-World Artificial Intelligence Applications

The possibilities of artificial intelligence are more abundant than people realize. For example, people in real life interact with cyber assistants on their favorite shopping websites, request Facebook to create an advertisement for the promotion of a business, direct Alexa to play their favorite songs, ask Google about the direction of some desired place (Braga and Logan, 2017). These are methods of using artificial intelligence to make life easier and furnish more to consumers. The following are some more:

- Amazon furnishes transactional artificial intelligence with algorithms that continuously become more progressed. Presently, it can forecast people's buying habits and give information about the products.
- Pandora's musical DNA procedure utilizes more than 500 musical characteristics from songs that experienced musicians have humanly searched to suggest the latest songs to users according to their choices.
- “Nest” is a thermostat that is “voice-controlled by Alexa” according to preferred heating or cooling temperature (Gabriel, 2020).

### Artificial Intelligence Advantages

Artificial intelligence attaches value to computer systems' existent capabilities by continuously and accurately furnishing digital information and tasks (Hagras, 2018). Artificial intelligence can support improvement through:

- Less human error and fewer mistakes
- Enhanced business decisions with an approach to actual-time data
- Automatic tasks and procedures
- Enhanced operational efficiency and productivity
- Quality-lead generation
- Enhanced data learning with improved access to large data pools
- Enhanced service with consumer knowledge (Wirtz et al., 2019)

Moving into insights, the following are five elaborated ways artificial intelligence can work for a firm:

- **Data analysis and collection:** Artificial intelligence makes data analysis and collection budget-friendly, instinctive, and well-timed so the firm can automatically comprehend more about their consumers, safe repeats, and new establishments (Parson et al., 2019).
- **Smart hiring:** Machine learning algorithms can discover best practices for an organization's particular hiring requirements and generate a list of short-listed and best candidates (Wang, 2019).
- **Back-Office Efficiency:** Artificial intelligence can manage tasks such as scheduling, accounting, and some other day-to-day operations very quickly, without any mistakes (Došilović et al., 2018).
- **Customer Service:** Virtual consumer service representatives function 24/7 and can furnish help to existing and prospective customers without any human guidance or control (Siau and Wang, 2018).
- **Targeted Marketing:** Classifying and arranging all accessible data about the service or product is an artificial intelligence distinction- allowing the organization to focus on marketing that particularly highlights customers' requirements (Došilović et al., 2018).

## AI Commoditization

**Figure 1:**
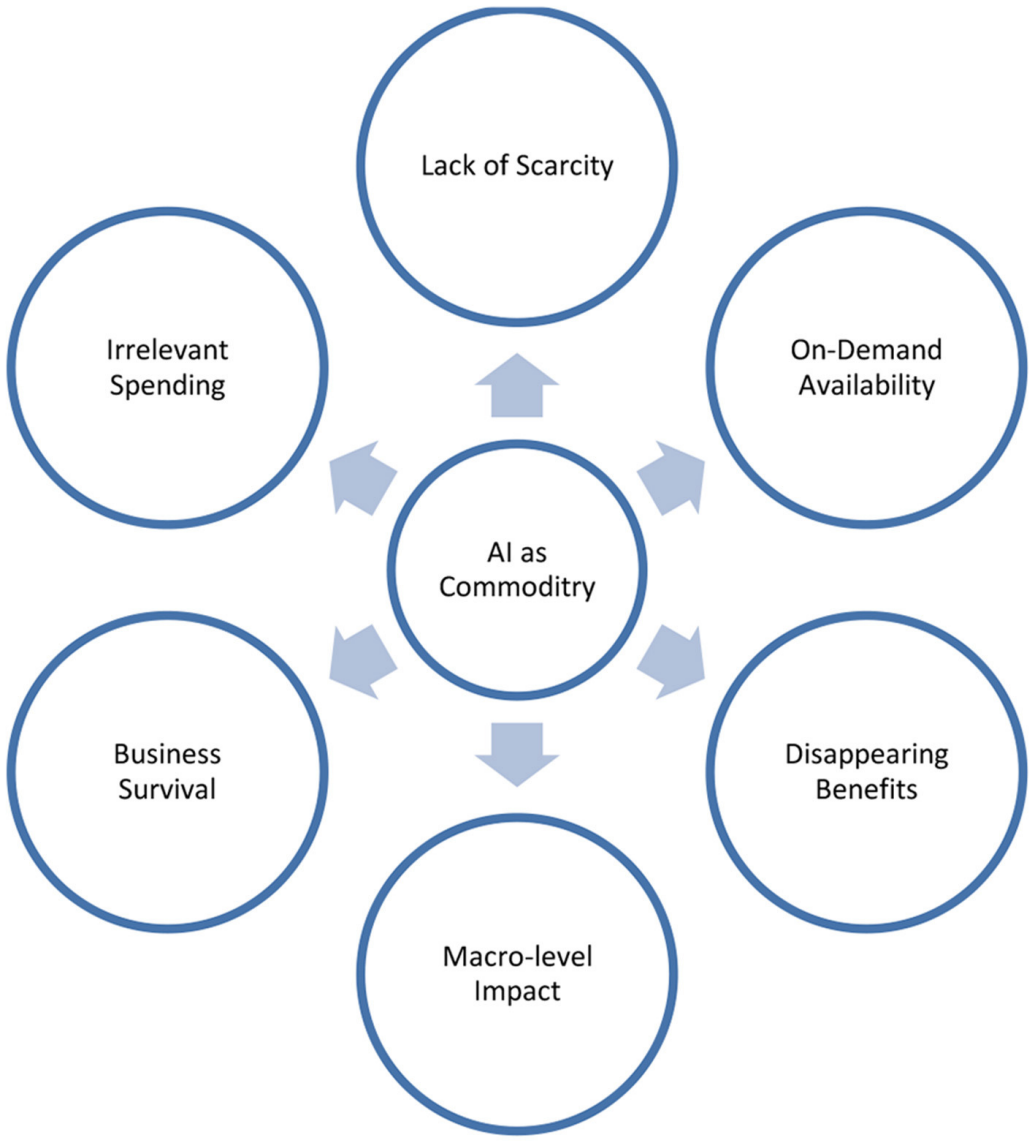
AI commodity model.

In his HBR article, Nicholas Carr argues that IT has become a commodity just like electricity, the telephone, the steam engine, the telegraph, and the railroad (Carr, 2003). Because of their very nature, commodities do not provide any strategic differentiation. Carr (2003) suggests that IT can be used to supplement and improve strategy implementation, but it is not the foundation of competitive advantage. The research poses the question: “*Does the commoditization of IT apply to artificial intelligence?*” The argument is that there is emerging evidence that the answer is “yes.” The argument is given and based on that AI commodity model is shown in Figure 1 below.

### Argument 1- AI Scarcity Is Vanishing

Many firms have created C-level positions for technology managers. Some organizations have appointed strategy consulting companies to identify how to take advantage of their artificial intelligence investments for strategic differentiation (Wilner, 2018). The simple supposition, the increased strategic value of AI must match its degree of effectiveness and pervasiveness. It is both a logical as well as an intuitive supposition. However, the supposition is incorrect. According to economic theory, what makes a resource strategic- what provides it with the capability to be the foundation for a continuous competitive edge- is not effectiveness and pervasiveness but scarcity. Machine learning, deep learning, and reinforced learning that form the basic building blocks of AI have become readily available commodities. Currently, AI solutions are privately owned for the most part. However, as it becomes increasingly available, accessible, and affordable, the competitive edge of being “AI-enabled” starts to dissolve. Essentially, if everyone is equipped with the same capabilities, then there is no competitive advantage anymore.

### Argument 2- AI On-Demand Availability

Many big cloud providers are offering AI services out of the box. Instead of requiring dedicated teams of data scientists, companies can purchase and consume AI on-demand as a service. These services avoid the complexity of building ML models and pre-requisite knowledge for domains such as speech recognition, text analytics, or image recognition, among others. Furthermore, as fully-managed services, these AI capabilities require no DevOps on the customer's part. Without oversimplifying, for speech-to-text, for instance, you need to upload audio files and click “transcribe.” Then retrieve the text output for whatever downstream analytics you would like to do on that text.

In the future, AI features will be built-in in all applications. In other words, we will see a convergence toward AI parity and performance, much like we see that email platforms are more-or-less the same, even if they are made by different companies (e.g., Hotmail, Gmail, Yahoo, AOL, etc.). In this way, AI will become standard and ordinary. But, ultimately, AI capability becomes a commodity. When a resource becomes vital to the competition but insignificant to strategy, the risks it generates become more significant than the benefits it delivers (Vähäkainu and Lehto, 2019). This implies that companies' differentiation (sustainable value) must be derived from something else—something proprietary, something not readily available to others.

### Argument 3- AI Disappearing Benefits

Many scholars have emphasized the importance of the integration of artificial intelligence, especially machine learning, in existing technologies. However, many comparisons have emphasized both economies and investment formats linked with the technologies-the “boom to bust cycle or the roles of artificial intelligence in re-structuring the overall operation of firms.” There is a significantly more minor discussion about the influence or no influence and competition at the company level for artificial intelligence.

### Argument 4- AI Macro-level Impact

Artificial intelligence appears to have had significant market changes. With the advancement and evolution of artificial intelligence, the ways of living and working are also transforming, but it also raises an inevitable question about the impact of artificial intelligence on the economy, customers, and businesses. Employees want to get information about what changes artificial intelligence can bring to their income and job. In contrast, companies are concerned about how they can gain benefits from the opportunities that artificial intelligence offer and which areas need more investment. After all these considerations, the main question is how to create artificial intelligence so that it is transparent and responsible enough to gain the trust of customers and business stakeholders. Even without the elevation in labor demand due to economic factors, artificial intelligence will need new roles and jobs. In addition, with the jobs in the application and development of artificial intelligence, the technologies will need to be created, operated, maintained, and regulated.

### Argument 5- AI Is Becoming Vital to Business Survival

When a system becomes vital to the competition but insignificant to strategy, the risks it generates become more than the benefits it furnishes. Consider what happened since the introduction of computers. No firm develops its strategy around its computer usage in today's world, but even a shortage of systems can cause devastation. The operational risks have created an association with artificial intelligence- technical defects, discontinuance, service interruption, undependable partners or dealers, security vulnerability, terrorism- and some have become amplified as firms have shifted from strictly controlled artificial intelligence systems to shared, open ones. In today's world, a disturbance in an information technology system can completely paralyze the company's ability to make and deliver its products; the disruption in artificial intelligence systems can negatively affect customers' connection and build a negative reputation (Kaplan and Haenlein, 2020). Still, some firms have performed an efficient job of recognizing and moderating their vulnerabilities. Panicking about what might go in the wrong direction may not be as alluring a job as hypothesizing about the future but is the more important job at present. In the distant future, even though the most considerable artificial intelligence risk facing most firms is more unimaginative than devastation. It is clearly, excessive spending (Huang and Rust, 2018). Artificial intelligence may be a product, and its costs may decrease swiftly enough to guarantee that any new skills are quickly shared, but the very real fact that so many firms are interlinked with so many firm operations means that it will commence using a massive amount of collective spending. For many firms, just remaining in the market will result in huge artificial intelligence expenditure (Kim, 2020). What is significant- and this remains true for any product output-is the ability to separate vital investments from those that are permissive, avoidable, and even inefficacious. At an upper level, more robust cost management needs more diligence in assessing anticipated returns for the investments of systems, more innovation in investigating cheaper and simpler replacements, and a broader directness to externalization and other partnerships. Nevertheless, many firms can also derive vital savings by clearly eliminating waste. Personal computer systems are an excellent example (Cockburn et al., 2018).

### Argument 6- AI Spending Is Irrelevant

Each year, firms buy more than 115 million personal computers, most of which substitute previous models. The vast majority of employees who utilize personal computers depend on only some simple applications- web-browsing, email, word processing, and spreadsheets. These applications have been technologically intelligent for years, and they need only some fragment of the computing power furnished by the microprocessors in today's world. Nonetheless, firms spend substantial amounts across the board on software and hardware updates (Townsend and Hunt, 2019). Much of that investment is compelled by the strategies of vendors. Huge software and hardware distributors have become very efficient at delivering the latest artificial intelligence capabilities and features in ways that force firms into purchasing the latest computers, networking equipment, and applications much more regularly than required. The time has arrived for artificial intelligence system buyers to endorse and confer contracts that ensure their computer investments' durable effectiveness and force rigid restrictions on reforming costs. If vendors resist, firms should be ready to find cheaper solutions involving open-source apps and essential elements that network personal computers, even if they give up on features. If a firm requires proof of the type of money that could be saved, it requires only a check at Microsoft's profit margin (Vähäkainu and Lehto, 2019).

Additionally, to be compliant in their buying, the firms have not efficiently utilized artificial intelligence. That is precisely the case with Big Data, accounting for more than half of many firms' artificial intelligence expenditures (Duan et al., 2019). A large quantity of the stored data on business networks has significantly less to do with production or serving consumers- it involves emails and files, including video clips and MP3s. Artificial intelligence's world assesses that as much as 80% of a regular Windows network is famished, which is an unnecessary expense for firms (Carter and Nielsen, 2017). Limiting staff ability to save files randomly and continually may look objectionable to various managers, but it can create an actual effect on the bottom line. Now that artificial intelligence has become the principal capital expense for most firms, there is no reason for waste and negligence (Floridi, 2020). Given the swift momentum of technological advancement, delaying artificial intelligence investments can be another solid way to dimmish costs, decreasing the company's opportunity to be burdened with problematic or soon-to-be-archaic technology. Many firms, specifically during the 2000s, boosted their artificial intelligence investments because they desired to gain an advantage at first or because they had a fear of being left behind. Except in some scenarios, both the desire and fear were indefensible (Greene et al., 2019). Some firms may be distressed that being niggardly with AI dollars will deface their competitive positions in the market. However, various studies show that businesses that integrate AI spending continuously have more significant expenditures which infrequently convert into higher financial outcomes. The contradiction is true. The most immoderate payers seldom bring the best results. Many firms spend too much on AI but get significantly less in response (Russell, 2017).

Based on the above arguments, a commoditization model is devised that shows the leading factors of commoditization.

**Figure 2:**
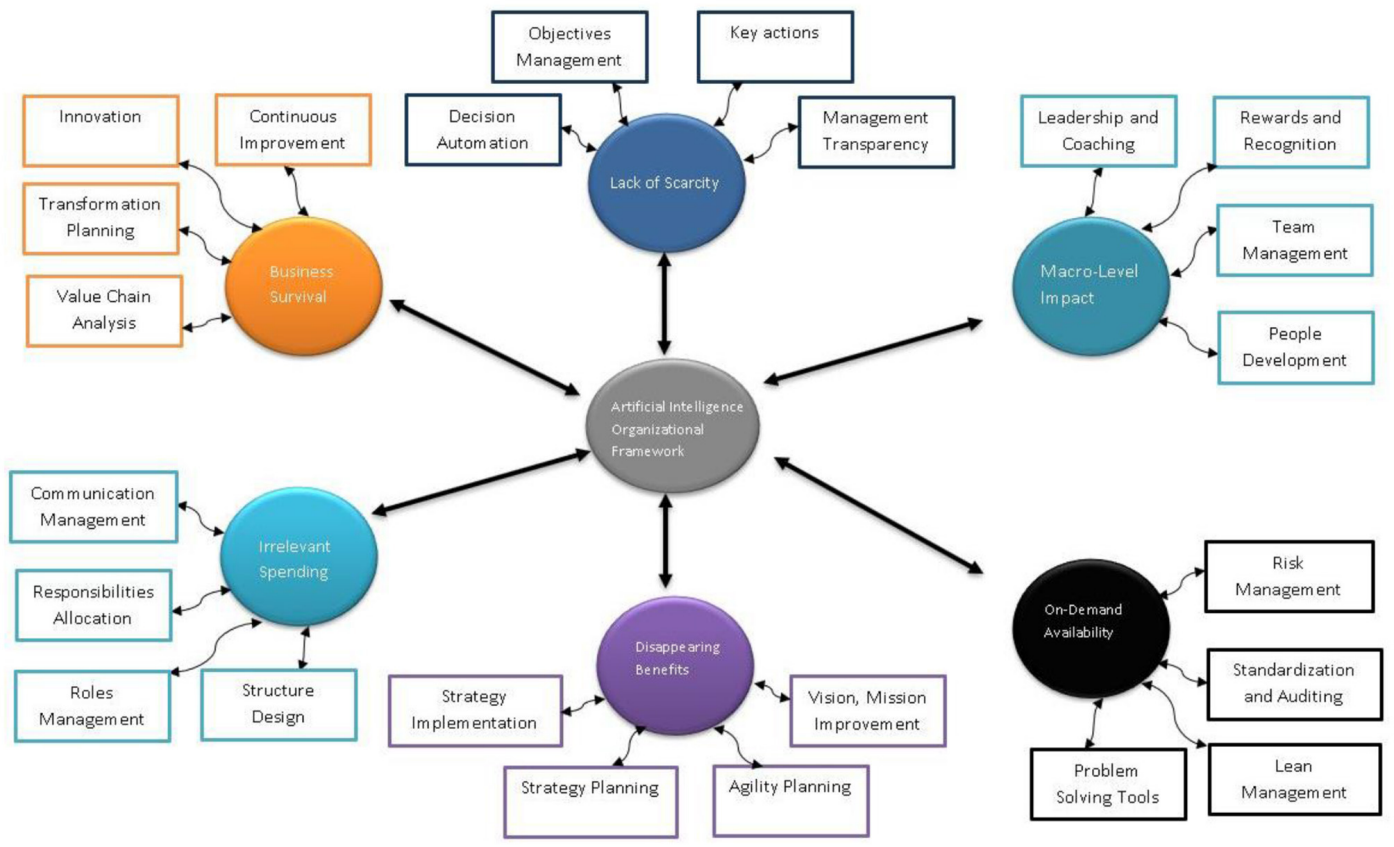
Artificial intelligence organizational framework.

To manage the commoditization issue, an artificial intelligence-based organizational framework is proposed that can help to add value for organizations facing issues due to commoditization of artificial intelligence as shown in Figure 2.

### AI Organizational Framework

The following provides the overview of each phase of the artificial intelligence organization framework and its sub-components.

### Macro Level Impact

#### Leadership and Coaching

When conjoined with an actual life-experienced teacher or coach, two vital types of artificial intelligence are now being adopted to assist learners to practice actual-world skills in their work as the latest form of performance support such as deep learning and expert systems.

#### Rewards and Recognition

By any means, recognition and reward program owners should be responsible for applying and understanding advanced analytics and artificial intelligence to the sector of human motivation, performance, and engagement.

#### Team Management

With the adoption of chatbots and natural language processing, artificial intelligence can recognize who requires positive feedback and let the employer know or give the feedback. It can also identify who requires more training and make a fact-filled meeting schedule for the managers and employees.

#### People Development

Eventually, implementing artificial intelligence in training, development, and learning will let people gain content-based training according to their skills, traits, and preferences. First, however, artificial intelligence needs to create programs attainable to people even with various kinds of disabilities.

### On-Demand Availability

#### Risk Management

Artificial intelligence can benefit risk management in different areas. For example, artificial intelligence can permit risk managers to respond quickly to the latest and evolving exposures, from the capability to process massive amounts of data to automate some repetitive and arduous risk management stages.

#### Standardization and Auditing

Auditing and standardization can be transformed with machine learning, heading to enhanced accuracy and productivity. With the use of machine learning, auditing firms can automate massive volumes of data to recognize incongruities and risky transactions that humans can analyze later.

#### Lean Management

Enhancing efficiencies and eliminating waste is a principle of artificial intelligence and also of lean management. Artificial intelligence and lean management can be revolutionary as firm leaders integrate employee experience in creating novel roles and technological structures.

#### Problem-Solving Tools

Artificial intelligence can solve problems by performing differential equations, logical algorithms, and using polynomial equations, and completing them utilizing modeling paradigms. As a result, there can be different solutions to one problem, which various heuristics attained (Iphofen and Kritikos, 2019).

### Disappearing Benefits

#### Vision, Mission Improvement

Artificial intelligence is an emerging technology and may face unpredicted challenges; many firms want to take the risk and adopt artificial intelligence technology. Some of the main objectives are to lessen the operational costs, improve customer experience, and upsurge revenue (Ramamoorthy and Yampolskiy, 2018).

#### Agility Planning

The present era's competitive environment is in a condition of continuous acceleration, and it is dubious about slowing down ever. The breadth of technology, pace, and scale modification in previous years has developed such productive ground for innovation that it has essentially modified the ways firms succeed. As a result, the firms winning in today's world are the most agile, forecasting and responding to the change faster (Petrović, 2018).

#### Strategy Planning

Artificial intelligence has a significant impact on firms and plays an essential role in management. Artificial intelligence modifies the ways of business management and strategic planning. It helps the firms attain a competitive edge in the market and helps them achieve great success.

#### Strategy Implementation

Any artificial intelligence strategy will work as an unceasingly evolving parameter to confirm the selected artificial intelligence programs are created and function to business objectives, integrating innovation in all business functions. For the transition in the data-driven or at an increased level of artificial intelligence-driven solutions, firms will have to implement a culture of experimentation inclination and continuous enhancement by starting small and applying short and incremental cycles, evolving true artificial intelligence revolution over time.

### Irrelevant Spending

#### Structure Design

Organizations can use artificial intelligence to monitor the conflicting issues in the organization's structure that will take into account the capabilities, strategy, and unique characteristics. As a result, it will minimize irrelevant spending and increase growth and profitability.

#### Roles Management

Artificial intelligence in firms is an advancement that can allow managers to become excellent. Artificial intelligence can be used in many facets, from enhancing relationships with staff and consumers to distinguishing patterns in excessive data volume to repetitive tasks.

#### Responsibilities Allocation

Responsibilities allocation is one of the secrets to boosting organizational benefits by managing as many tasks as possible. Numerous computational multi-agent systems utilize the capability of agents for responsibilities allocation.

#### Communication Management

Artificial intelligence has powered chatbots to automate and manage communication, and they are substitutes for dealing with humans. These chatbots can control communication in various ways to engage with consumers, for example, responding to queries or providing assistance.

### Business Survival

#### Value Chain Analysis

Using artificial intelligence in value chain analysis, managers can improve their decision-making procedures by forecasting unexpected abnormalities, building up bottlenecks, and finding solutions to restructuring manufacturing schedules that tend to be increasingly inconstant because of dependencies in production operations.

#### Transformation Planning

Incredible precision can be attained through transformation planning by implementing artificial intelligence using deep neural networks. Furthermore, it helps to get the most out of data by utilizing the latest learning algorithms, proving it is a flexible technology (Smith, 2019).

#### Innovation

Artificial intelligence and innovation together can enhance many business areas. For example, in customer service, chatbots are now communicating with online consumers to improve customer service. In addition, artificial intelligence is being utilized for the HR department to accelerate the recruitment procedure, and for the marketing department, artificial intelligence-powered tools are used to customize the consumer experience (Liu, 2018).

#### Continuous Improvement

Using artificial intelligence, systems can check thousands of mathematical models of manufacturing and outcome possibilities and be more accurate about the analysis during the adoption of new information, for example, new products, supply chain disruptions, and unexpected changes in demand. Thus, it helps in continuous improvement for firms to attain a competitive edge in the market.

### Lack of Scarcity

#### Decision Automation

Artificial intelligence can boost human intelligence and allow intelligent decision-making. It helps in the detection of wrong decisions and accelerates the whole decision-making procedure. Artificial intelligence enables the automation of decision-making without human interference (Mozer et al., 2019).

#### Objectives Management

Artificial intelligence has become an essential facet for many firms. It helps in many tasks in the firm and aids in modernizing business procedures and objective management, supporting the firm to perform more efficiently and achieve the firm's objectives more proficiently.

#### Key Actions

Many firms focus on the outcomes of artificial intelligence. For firms concerned with minute details, there are four key actions to recognize: collaborative filtering, categorization, machine learning, and classification. These four key actions also signify the steps of the analytical procedure (Lutz, 2019).

#### Management Transparency

Management transparency is the way firms and leaders behave and think. The firms using artificial intelligence require more openness, accountability, and communication between employees and managers (Lui and Lamb, 2018).

## Are We Ready For AI?

Artificial intelligence is changing the rules of competition within industries worldwide. Opportunities associated with AI are considered the most important technological growth regarding its vast potential for adding business value and competitive advantage (Miailhe and Hodes, 2017).

AI applications and adoption offer each business entity as many new challenges as it does opportunities. AI technology has already transformed businesses everywhere, small or large, developed or start-ups. AI has the potential to level the playing field. However, it is essential to understand the other factors that will help drive successful AI capabilities. These include cognitive-based technologies like machine learning, natural language processing, and robotics. Those cognitive-based technologies will ultimately have a tremendous impact on every business level. It is acknowledged that a proper understanding of the issue and keen insights into this change is not an option. It is “a must.”

It raises a critical question that will be addressed in this section, the question is “***Are we ready for AI?”***

In light of IT Masters definition appraised in Westerman et al. (2014), and to answer the question “Are we ready for AI?,” we focus on two different AI application scenarios found in today's business organizations. First, Westerman refers to organizations that apply AI technologies seamlessly to every aspect of their businesses as “AI Masters.” They know exactly where and how to invest in AI opportunities, knowing the impact of those investments. AI Masters can see AI as the best way to increase business value, increase efficiency gains, and gain a competitive advantage. It could be achieved by applying AI solutions that ultimately impact customer relations, customer engagements, internal/external business operations, customer expectations, and even business models. AI Masters deeply understand the implications of evolving AI-driven automation ecosystems far beyond narrow AI applications. Accordingly, AI is not limited to changing how business works. It is also fundamentally transforming the traditional thinking and meaning of innovation.

Some organizations adopt new AI technologies without having a real strategy and without fully determining how AI technologies will be integrated within the organization. As a result, those organizations invest large amounts in their AI solutions. However, because they lack a strong and clear vision of the future, they will waste most of what they have invested. In this scenario, organizations have already invested in AI applications such as robots, AI power assistants such as virtual assistant shoppers and chatbots, fraud preventions, etc. However, they lack strong leadership capabilities, which lead to the definite inability to realize the concrete benefits of their AI investments.

It is widely acknowledged that “replacing a man with a machine” is not a fashion that every organization should follow, bearing in mind that “*why to replace*” is as important as “*how to replace*.” These organizations tend to be mature in answering the second question but immature in answering the first one question. It leads to a narrowly overlaid AI adoption strategy as they mainly focus on using AI to change the way they provide the services without understanding why to they need to change it. In this way, they fail to answer questions such as why analyze data? Why predict performance? And why transform? As a result, the AI trend in applications in this scenario does not respond adequately to rapidly evolving intelligence capability. It will negatively affect their understanding of the broader AI trends on the horizon and their future AI preparations.

## Summary of Findings and Conclusion

Artificial intelligence is becoming a commodity as more solutions are readily available for the end-users. This paper addressed the important aspects of AI as a commodity and provided a closer look at the various perspectives on the usage of AI in various fields. The findings show that AI commoditization is in the near future and must be avoided to sustain strategic differentiation. Otherwise, AI will have the same fate as previous technologies, i.e., information technology. The arguments on AI commoditization have been discussed in detail to show the leading factors of commoditization. Compiling leadership and AI capabilities will achieve greater performance than either dimension can deliver on its own. The organizations that excelled in AI capability and leadership capability may have higher financial outcomes. More relevant and rigorous studies are needed to shed light on the importance of improving the organizations' AI-focused leadership capabilities and how to enhance the business model to adapt the AI application, bearing in mind that the inability of the organizations to enhance their leadership/AI technical capabilities will lead to ultimate failure and unwanted consequences. The previous discussion has drawn us to define some other parameters that may affect AI application success, mainly the leadership capability that is as important as the AI technological capability.

Good AI leadership capability is the lever that uses AI technology for real business transformation. They do not apply the “bottom-up” AI applications model. Instead, they have a very strong “top-down” leadership model by setting the AI directions, building momentum, measuring the initiatives, and ensuring that the organizations can follow through. More specifically, the top-down leadership model occurs by setting up clear AI goals, then engaging their employees by energizing them to drive through the AI journey. Leaders know what they are aiming for. Even though they believe in the workforce as one key asset in their organizations, at the same time, they know exactly when “replacing a machine with a human” should happen. The AI commodity model links the arguments with the organizational framework used to add value for the organizations facing the commoditization issue. The AI-based organizational framework addressed the value added to each phase of the commodity model. The framework can be used to drill down to the possible options available for organizations and end-users to have a clear pathway for the usage of AI responsibly. The history and arguments validate that shortly the value of AI solutions will not impact businesses. For this purpose, an artificial intelligence-based organizational framework is proposed that provides an overview of value-added features that can help organizations lower the impact of AI commoditization.

## Author Contributions

MT worked on the conception and design of the idea which was given by AA. MT and AA worked on the writing literature review, perspectives, and overall organization of the paper. SS worked on the technical aspects of artificial intelligence and machine learning. All three worked together on writing different sections of the paper and contributed to read, revise, and approved the submitted version.

## Conflict of Interest

The authors declare that the research was conducted in the absence of any commercial or financial relationships that could be construed as a potential conflict of interest.

## Publisher's Note

All claims expressed in this article are solely those of the authors and do not necessarily represent those of their affiliated organizations, or those of the publisher, the editors and the reviewers. Any product that may be evaluated in this article, or claim that may be made by its manufacturer, is not guaranteed or endorsed by the publisher.
